# The gephyrin scaffold modulates cortical layer 2/3 pyramidal neuron responsiveness to single whisker stimulation

**DOI:** 10.1038/s41598-024-54720-7

**Published:** 2024-02-20

**Authors:** Yuan-Chen Tsai, Mohammad Hleihil, Kanako Otomo, Andrin Abegg, Anna Cavaccini, Patrizia Panzanelli, Teresa Cramer, Kim David Ferrari, Matthew J. P. Barrett, Giovanna Bosshard, Theofanis Karayannis, Bruno Weber, Shiva K. Tyagarajan, Jillian L. Stobart

**Affiliations:** 1 Institute of Pharmacology and Toxicology, University of Zurich, Winterthurerstrasse 190, 8057 Zurich, Switzerland; 2 Center for Neuroscience Zurich (ZNZ), Winterthurerstrasse 190, 8057 Zurich, Switzerland; 3 Brain Research Institute, University of Zurich, Winterthurerstrasse 190, 8057 Zurich, Switzerland; 4 Department of Neuroscience Rita Levi Montalcini, University of Turin, Turin, Italy; 5 College of Pharmacy, University of Manitoba, Winnipeg, MB R3E 0T5 Canada

**Keywords:** GABA_A_ receptors, Gephyrin, Barrel cortex, Electrophysiology, Two-photon calcium imaging, Molecular biology, Neuroscience

## Abstract

Gephyrin is the main scaffolding protein at inhibitory postsynaptic sites, and its clusters are the signaling hubs where several molecular pathways converge. Post-translational modifications (PTMs) of gephyrin alter GABA_A_ receptor clustering at the synapse, but it is unclear how this affects neuronal activity at the circuit level. We assessed the contribution of gephyrin PTMs to microcircuit activity in the mouse barrel cortex by slice electrophysiology and in vivo two-photon calcium imaging of layer 2/3 (L2/3) pyramidal cells during single-whisker stimulation. Our results suggest that, depending on the type of gephyrin PTM, the neuronal activities of L2/3 pyramidal neurons can be differentially modulated, leading to changes in the size of the neuronal population responding to the single-whisker stimulation. Furthermore, we show that gephyrin PTMs have their preference for selecting synaptic GABA_A_ receptor subunits. Our results identify an important role of gephyrin and GABAergic postsynaptic sites for cortical microcircuit function during sensory stimulation.

## Introduction

GABAergic neurotransmission plays a crucial role in controlling cortical microcircuit activity^1^ by coordinating pyramidal neuron activity during sensorimotor behavior in a synchronized manner^2^. Several subtypes of GABAergic interneurons make up approximately 20% of all cortical neurons. Of these subtypes, parvalbumin-expressing (PV+) fast spiking interneurons typically synapse onto the somata and proximal dendrites of pyramidal cells and are activated during passive and active whisking. In contrast, somatostatin-expressing (SOM+) interneurons typically synapse onto distal dendrites of pyramidal cells and become hyperpolarized during whisking^3^. The importance of inhibition is even more prominent in awake mice as the cortical state changes from quiet to active whisker behavior, and this involves the reorganization of GABAergic neuronal network activity^4^. The research on GABAergic neurotransmission in cortical microcircuits focuses mainly on the interneuron subtypes, and significant knowledge gaps persist in the contribution of GABAergic postsynaptic sites in regulating neuronal and microcircuit activities.

The recruitment of specific GABA_A_ receptor (GABA_A_Rs) subtypes (α1, α2, and α3) to synaptic locations is facilitated by diverse receptor-interacting proteins that include neuroligin-2^5^, collybistin^6,7^, and gephyrin^8^. Among these proteins, gephyrin is unique because it aids activity-dependent adaptations at GABAergic postsynaptic sites over different time scales, depending on its post-translational modifications (PTMs)^9^. Gephyrin is phosphorylated by diverse kinases^10^, such as S303 phosphorylation by protein kinase A (PKA) and S305 phosphorylation by calcium calmodulin kinase II α (CaMKIIα). Phosphorylation at S303 and S305 sites promotes gephyrin scaffold recruitment, increasing a2-GABA_A_Rs availability at inhibitory synapses^11^. The phosphorylation at these sites is NMDA receptor and activity-dependent, in turn increasing GABAergic neurotransmission^12,13^. SUMOylation, a type of PTM made by forming covalent bonds between SUMO proteins and the lysine residues, was also found to affect gephyrin at K148 and K724. Together with SUMOylation, the acetylation at K666 prevents gephyrin clustering and reduces GABAergic transmission^14^. Gephyrin is also palmitoylated and nitrosylated inhibiting its scaffolding function^15,16^. Super-resolution microscopy studies of gephyrin PTM mutants have shown that compaction of the gephyrin molecule and synaptic dwell time of α2-GABA_A_Rs contribute to activity-dependent adaptation^9,11^. However, the functional significance of gephyrin clusters and their PTMs in vivo at the microcircuit level, particularly during the encoding of sensory information, remains unexplored.

To investigate the role of the gephyrin scaffold and gephyrin PTMs in cortical microcircuit activity, we employed the mouse whisker-barrel cortex system. The primary somatosensory cortex (S1) encodes passive sensory experience, training on sensory tasks, and perceptual learning^17^, and these tasks rely heavily on the harmony between excitatory and inhibitory activity in the cortical microcircuits. Within S1, the rodent barrel system is a well-established model to study sensory perception, and sensory information is processed in a selective manner via sparse encoding in the L2/3 barrel cortex. For example, whisker deflection leads to depolarization of neurons, but only around 10% of cells respond with action potentials^18^. This phenomenon is due to strong inhibition from GABAergic interneurons that are well connected to the excitatory neurons in L2/3 barrel cortex. The probability of finding excitatory-excitatory connections between two pyramidal neurons in supragranular layers is relatively low, whereas the connectivity between pyramidal cells and inhibitory interneurons is much higher^1,19–21^. The excitability of a cortical microcircuit is tightly modulated by GABAergic inhibition in the L2/3 barrel cortex^22^, making it an ideal system the examine the functional role of gephyrin in a microcircuit. In this study, we expressed gephyrin mutant variants, which lack specific PTMs, in L2/3 pyramidal neurons and measured their impact on inhibitory currents and neuronal population activity changes during whisker stimulation. Our results indicate that different gephyrin PTMs change microcircuit activity, possibly by promoting clustering or confinement of GABA_A_Rs, and have the potential to fine-tune L2/3 excitability at the single-cell and population levels.

## Materials and methods

All experiments were performed in accordance with the European Community Council Directives of November 24, 1986 (86/609/EEC) and approved by the cantonal veterinary office of Zurich (ZH056/2018). We followed ARRIVE 2.0 guidelines when reporting animal data in this study.

### Animals

Female mice C57BL/6 J were purchased (Charles River, Germany). They were group-housed in an inverted 12-h light/dark cycle, and they were randomly assigned to each gephyrin group. All mice underwent surgery at 8–12 weeks of age and were imaged repeatedly (2–4 times per week) under a two-photon microscope for up to 5 months.

### Cloning and virus production

The AAV2/6-CaMKIIα-RCaMP1.07 construct was generated by cloning of the RCaMP1.07 gene^23,24^ into an adeno-associated plasmid backbone (AAV2) under a calcium/calmodulin dependent protein kinase II alpha (CaMKIIα) promoter.

The eGFP-gephyrin-WT, eGFP-gephyrin-K148R, eGFP-gephyrin-DN, eGFP-gephyrin S303/305A, and eGFP-gephyrin expression vectors have been described previously^11,14^, and were subcloned into an AAV2 plasmid backbone containing the human synapsin1 (hSyn1) promoter, in an inverted orientation, and flanked by 2 different loxP sites. The transgene was packaged into AAV 6 serotype. All the AAV6 recombinant viruses were generated by the Viral Vector Core at the University of Zürich. AAV2/6-CaMKIIaα-CreER^T2^ virus was purchased from Vector Biolabs (#2014–1208).

### Tamoxifen administration

Tamoxifen (1 mg per animal; Sigma, T5648) was given intraperitoneally (i.p.) for 4 consecutive days to induce Cre recombinase activity from CreER^T2^. The neurons expressing the transgene were imaged 5–7 days post final tamoxifen injection.

### Surgery and virus injections for two-photon Ca^2+^ imaging

Surgical procedures were divided into two steps, which were 2–4 days apart. In the first surgery, after fixing the mouse head in a stable position in a stereotaxic frame, an incision was made along the mid-line to expose the skull. After cleaning the bone, bonding reagent (ONE COAT 7 Universal, Coltene) was applied, and then a head cap was created using layers of light-cured dental cement (SYNERGEY D6 Flow, Coltene). Finally, a custom-made aluminum head post was attached to the head cap. These procedures were carried out under isoflurane (4% for induction and 1.5–2% for maintenance, Forene, AbbVie). The second surgery involved a craniotomy, cortical viral injection and chronic window implantation. With a dental drill, a small piece of skull was removed above the sensory cortex to expose the barrel cortex. A glass pipette and hydraulic pump were used to inject 150 nL of virus (injecting speed 50–70 nL per minute) at a depth of 350 µm beneath the brain surface into the whisker areas identified by Intrinsic optical imaging (see below). Immediately after the injections, a 3 × 3 mm coverslip was fixed right above the exposed brain and secured with dental cement to the head cap. Buprenorphine (Temgesic 0.1 mg/kg) was given before and after surgical procedures for 3 days.

### Intrinsic optical imaging (IOI)

IOI was used to identify barrel areas of the corresponding whiskers in the left somatosensory cortex. This technique was used to image activation of barrel areas through the skull (before craniotomy) to map the whisker field for potential viral injections, and through the cranial window to map specific whisker areas before two-photon imaging. Under a red light (630 nm illumination), the activated brain region (imaged 400 µm under cortical surface) was identified by increased light absorption following whisker deflection by a piezo stimulator at 10 Hz for six seconds. Imaging was done by using a 12-bit CCD camera (Pixelfly VGA, PCO imaging), and the animals were maintained under 1–1.2% isoflurane.

### Two-photon imaging

A custom-built two-photon laser-scanning microscope^25^ with a 20× water immersion objective (W Plan-Apochromat 20x/1.0 DIC VIS-IR, Zeiss) was used for in vivo Ca^2+^ imaging in anaesthetized mice. The microscope was equipped with a Ti:sapphire laser (Mai Tai; Spectra-Physics) set to 1040 nm to excite RCaMP1.07. Fluorescence emission was detected with GaAsP photo-multiplier modules (Hamamatsu Photonics) fitted with a 520/50 nm band pass filter or a 607/70 band pass filter and separated by 560 nm dichroic mirror (BrightLine; Semrock). A customized version of ScanImage (r3.8.1; Janelia Research Campus) was used for setting imaging parameters and to control the microscope.

Confirmation of viral expression at the injected site and neuronal activation during whisker stimulation was also examined before the series of imaging sessions. A whisker was placed in the glass capillary attached to the piezo element (T223-H4CL-303X; Piezo Systems), and the capillary tube was deflected in 1.72° pulses at 90 Hz for 1 s, reaching speeds up to 648°/s^25^. The deflection occurred in the anterior–posterior direction, and this whisker stimulation paradigm was chosen because it has previously been reported to give reliable long-term responses in isoflurane-anesthetized mice^25^. Every imaging session comprised 40 trials of spontaneous activity and 40 trials with whisker deflection, and the duration of each trial was 15 s (inter-trial interval: 3 s). Fast images were taken (11.84 Hz, 128 × 128 pixels) to capture neuronal Ca^2+^ responses. Imaging depth ranged from 160 to 200 µm beneath the cortical surface, which was in layer 2/3 of the barrel cortex. Once a field of view was selected, the same field was imaged for 3–4 sessions on different days, and an extra 3 sessions if the animals were subjected to tamoxifen injections.

### Confocal imaging

Fixed brain slice tissue was prepared at the end of the two-photon study (i.e. more than 7 days after the last tamoxifen injection) for immunohistochemistry (antibodies listed in Table 1). Images were taken from coronal sections of the virus-injected mice using a 40X objective (numerical aperture 1.4) and confocal laser scanning microscopy system (LSM 700, Carl Zeiss). The pinhole size during acquisition was set to 1 Airy unit. The somatosensory cortex were imaged as a z-stack with 4 sections separated by a 0.5 µm step. All imaging parameters were kept constant between control and experimental groups. Representative images were adjusted in contrast and brightness using ImageJ.

**Table 1:** Antibodies used in this study.

Target	Distributor/produce from	Description and Ref. no.	Dilution	Marker
GFP	Aves labs	Chicken, GFP-1020	1:5000	IHC
CaMKIIa	Thermo fisher scientific	Mouse, 13-7300	1:1000	IHC
GABRA1	Home-made^26,27^	Rabbit	1:10000	ICC
GABRA2	Home-made^26,28^	Guinea pig	1:2000	ICC

### Slice preparation

For miniature inhibitory postsynaptic currents (mIPSCs): 4 week old mice were injected in the barrel cortex with the described virus. After another 4 weeks, mice were sacrificed by cervical dislocation followed by decapitation under anaesthesia. The brains were removed quickly and immersed in ice-cold cutting solution (in mM): 110 sucrose, 60 NaCl, 3 KCl, 1.25 NaH2PO4, 28 NaHCO3, 0.5 CaCl2, 7 MgCl2, 5 D-glucose. The slices were incubated at 32 °C for > 30 min in ACSF, in mM: 125 NaCl, 2.5 KCl, 1.25 NaH2PO4, 26 NaHCO3, 25 D-glucose, 2.5 CaCl2, and 2 MgCl2. 300 µm coronal slices of the virus-injected area of the somatosensory cortex were made using a Leica VT1200 vibratome (Leica). After the recovery period, slices were maintained at RT with continuous perfusion of carbogenated ACSF (95% O2, 5% CO2). All recordings were performed at RT.

### Electrophysiology

For mIPSC: Recordings were amplified by Multiclamp 700B amplifier and digitized with Digidata 1440 (Molecular Devices). The recording electrode was pulled from a borosilicate glass pipette (3–5 MΩ) using an electrode puller (PC-100, NARISHIGE Group). Pipettes were filled with cesium-based solution containing (in mM): 120 CsCl, 10 EGTA, 10 HEPES pH 7.4, 4 MgCl2, 0.5 GTP and 2 ATP. Events were isolated by adding CNQX (25 µM, Merck), AP-5 (50 µM, alomone labs) and tetrodotoxin (1 µM, Affix Scientific). Cells were selected based on GFP expression, which was induced by tamoxifen administration. Ten min after establishing the whole-cell mode, mIPSCs were analyzed for a duration of 2 min. Events were recorded using Clampex 10.7 software (Molecular Devices). Recordings were filtered offline using a Bessel low pass filter at 500 Hz (Clampfit 10.7, Molecular Devices). Data were analyzed using MiniAnalysis 6.0.7 software (Synaptosoft) using the peak detection algorithm. The minimum threshold for event detection was set to twofold of the calculated baseline noise level for each recorded trace (4-10pA), as described previously^29^. In addition, events were included in the analysis only if the following criteria were established: (i) decay time > the rise time, (ii) rise time > 0.5 ms, and (iii) decay time > 1.5 ms. All detected events were further verified visually. Cell with unstable baseline, or a holding current less than − 400 pA or access resistance changing more than 30% between the beginning and the end of the recording were discarded. Measured and detected parameters were first averaged within each experiment and regrouped by condition. Statistical analysis were performed by ordinary One-way or Brown-Forsythe and Welch One-way ANOVA followed by Tukey’s or Dunnet T3 multiple comparison tests, respectively.

### Primary neuronal cultures, transfection and immunocytochemistry

Cortical culture on coverslips were prepared from rat (Envigo) embryos at E17. Plasmid transfections were done at 13 days in vitro (DIV 13) with 1 µg of total plasmid DNA per coverslip. The plasmid(s) were mixed with 2 µl lipofectamine 2000 (Thermo Fischer Scientific) and 1 µl (1:10 diluted) magnetofection beads (CombiMag, OZ Bioscience). The plasmid-lipofectamine-magnetofection mix were incubated at room temperature for 15 min before adding to neurons. The neuron dishes were placed on top of magnetic plates during the 45 min transfection in the 37 °C incubator. The coverslips were transferred to 1 ml conditioned media and returned to the incubator. The plasmids used in the groups are the following: pEGFPC2-gephyrin P1, pEGFPC2-gephyrin K148R, pEGFPC2-gephyrin DN, pEGFPC2-gephyrin S303A/S305A. After one-week of plasmid expression (DIV13+ 7), the coverslips were rinsed in ice cold PBS, incubated with primary antibody mix containing 10% normal goat serum, GABRA1 (rabbit) and GABRA2 (guinea pig) antibodies was added to the coverslips and incubated for 90 min inside a humidified chamber at 37℃ incubator (antibody details in Table 1). The live stainings with the GABRA1 and GABRA2 antibodies label synaptic receptor pools as puncta, and this method minimizes background staining coming from the intracellular compartment. The coverslips were rinsed in PBS at room temperature (RT), and fixed with 4% PFA for 10 min at RT. After rinsing out the PFA, the coverslips were incubated with corresponding secondary antibodies (see antibody list). Images from ~ 20 neurons (3 different batches) were analyzed for eGFP-gephyrin cluster colocalization with GABRA1 and GABRA2 containing GABA_A_Rs. Z-stack images (4 optical sections, 0.5 µm step size) were recorded of all sections using LSM 700 (Carl Zeiss). Images were taken using a 40 × objective with a numerical aperture of 1.4, and pixel size of 112 nm^2^. Cells from three independent experiments were pooled for analysis. For cluster analysis in primary neuron culture, a custom Python script using the ImageJ image-processing framework was used. The script can be used as a plugin and is openly available on a github repository (https://github.com/dcolam/Cluster-Analysis-Plugin). Representative example images were processed using ImageJ. One-way ANOVA was used to compare between different groups, with post-hoc Tukey test.

### Two-Photon quantification and statistical analysis

We conducted a sample size calculation for linear mixed models (smpsize_lmm) with the sjstats package in R (version 3.5.3). We input a moderate effect size of 0.2, a power of 0.8, and a significance level of 0.05. Cage mates were assigned to the same gephyrin mutant group. No animals were excluded from the study.

All individual neurons expressing RCaMP1.07 in a field of view were manually selected as regions of interest (ROIs) on ImageJ and further processed with custom-designed toolbox Cellular and Hemodynamic Image Processing Suite (CHIPS)^30^ for MATLAB (R2015b, MathWorks). The images for a field of view were first motion-corrected with a 2D convolution engine for x–y drift. For each manually selected ROI, the average fluorescence in the first 2.5 s was selected as the baseline (F) and the change in calcium fluorescence was calculated for each frame in the imaging trial (dF/F). Peaks were identified by the MATLAB findpeaks function following application of a digital band-pass filter (passband frequency of minimum 0.1 Hz and maximum 2 Hz) and a threshold of 5 standard deviations from the baseline. The peak onset time was calculated by the first time point of the smoothed signal trace (2 frames moving average) crossed over the threshold (the mean of the 2.5 s baseline plus 1.5 times the standard deviation) after the start of stimulation.

For the analysis, we focused on the 2-s time window indicated in Fig. 2A (1 s of whisker stimulation and 1 s after the stimulation). The amplitude, duration, and number of events were analyzed. The parameter “number of events” was defined as the number of events per cell in 40 trials, and this corresponded to the response probability. The neuronal population was further categorized into different responding groups based on their amplitude or number of events and presented as the percentage of the total population in each experimental group.

Statistical analysis was performed using R (version 3.5.3) with the multcomp package for linear-mixed effect models. We set the following as fixed effects which were tested individually or for their interactions: stimulation condition (with or without whisker stimulation), treatment with tamoxifen (TAM) (for the gephyrin mutant experiment, before or after tamoxifen injections), mutants (for gephyrin the mutant experiment, to compare between different mutants). Individual animal and ROIs were set as random effects. The data were presented with means (un-corrected) and standard error of the means (SEM). The *p* values reported for different comparisons were obtained by using a Tukey post-hoc test.

## Results

### Gephyrin scaffold dynamics modulate GABAergic neurotransmission

**Figure 1:**
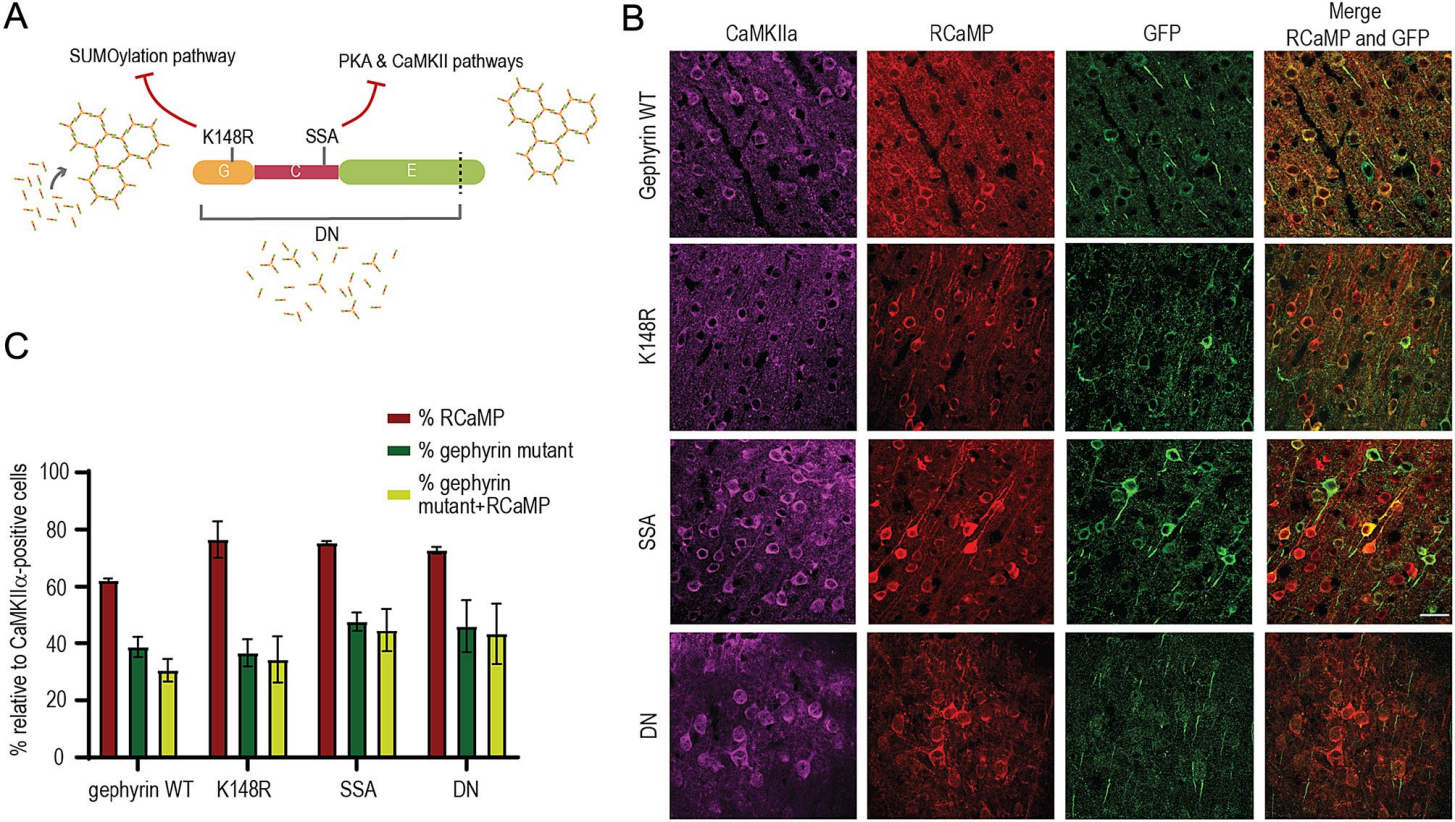
Co-expression of RCaMP and eGFP-gephyrin variants. (**A**) An illustration of different gephyrin mutations used in the study, along with the labels of signaling pathways that are affected by the mutated residue. The gephyrin-K148R (SUMO1 conjugation site mutant) facilitates scaffolding. The gephyrin-S303A and S305A (PKA and CaMKIIα phospho-null mutant) hamper NMDA receptor activity-induced scaling at GABAergic postsynaptic sites. The gephyrin-DN (lacks part of E domain) disrupts endogenous gephyrin scaffolds in neurons. (**B**) Representative images of neuron co-expressing RCaMP and eGFP-gephyrin variants after injection of AAVs in L2/3 barrel cortex in vivo. The following combination of viruses were injected into the barrel cortex (same combination used in 2P Ca^2+^ imaging): AAV6-hSyn1-flex-gephyrin variants, AAV6-CaMKIIα-CreER^T2^, AAV6-CaMKIIα-RCaMP1.07. All brain sections were stained for CaMKIIα and eGFP. Scale bar: 20 µm. (**C**) The averaged percentages of L2/3 pyramidal neurons from the field of views that were RCaMP-positive and RCaMP-positive + gephyrin variant-positive. Quantification of average expression of RCaMP-expressing neurons, gephyrin variant-expressing neurons, and neurons co-expressing RCaMP and eGFP-gephyrin mutants after normalization to total CaMKIIα-positive neurons.

We employed three different mutants that target distinct gephyrin PTMs. First, we used a SUMO1 conjugation-defective mutant (K148R) that increases sub-membrane gephyrin clusters^14^. Second, we utilized phosphorylation-null gephyrin mutants S303A/S305A (SSA) to block the activity-dependent recruitment of GABA_A_Rs to postsynaptic sites and alter the synaptic confinement of GABA_A_Rs^9^. Third, we employed the dominant-negative gephyrin mutant (DN), which lacks the last 12 amino acids and disrupts gephyrin scaffolding and GABAergic neurotransmission in vitro^14^ (Fig. 1A).

**Figure 2:**
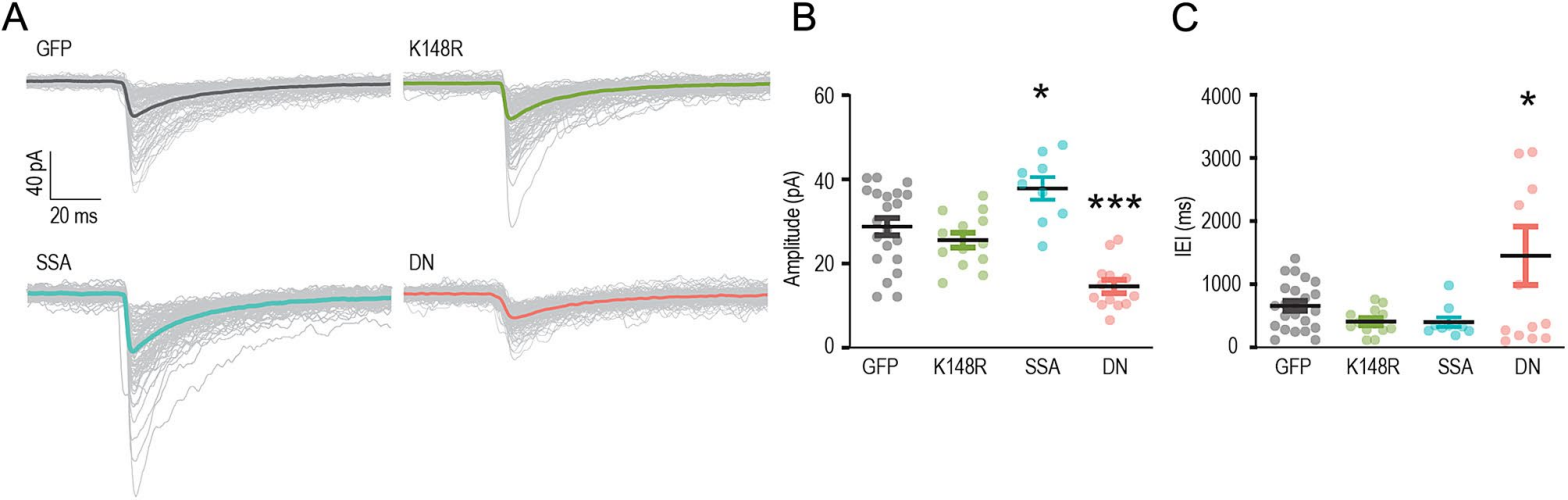
GABAergic neurotransmission affected by gephyrin mutant (**A**) Example traces from L2/3 pyramidal cells expressing gephyrin variants or GFP (control). Colored traces: averaged currents from one neuron. Grey traces: all events measured from one neuron. (**B**) Miniature inhibitory postsynaptic currents (mIPSCs) amplitude. (**C**) inter-event interval (IEI) in L2/3 pyramidal neurons expressing individual gephyrin variants. Data are reported as mean ± SEM. Statistics: eGFP, n = 23; K148R, n = 13; gephyrin-DN, n = 13; gephyrin-SSA, n = 10 neurons, from 4–5 mice in each group. One-way ANOVA with Tukey post-hoc test. Bars: mean ± SEM. *p ≤ 0.05, **p ≤ 0.01, ***p ≤ 0.001, ****p ≤ 0.0001.

**Figure 3:**
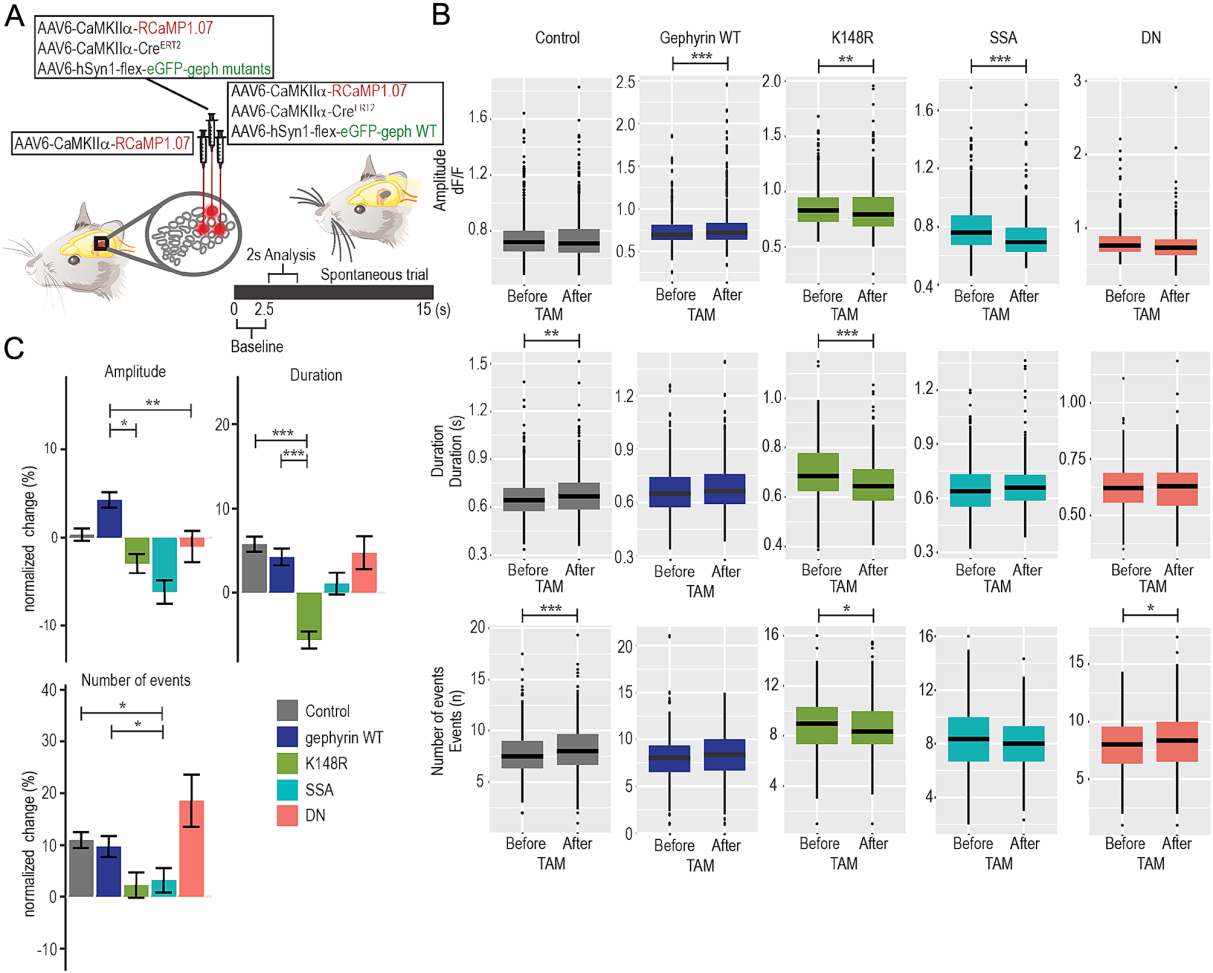
The expression of gephyrin mutants differentially modulates L2/3 pyramidal neuron spontaneous activity. (**A**) An overview of the viral infection in vivo. The control site received only AAV6-CaMKIIα-RCaMP1.07, while the experimental site received a combination of RCaMP/CaMKIIα- Cre/gephyrin flex viruses. (**B**) Absolute values for calcium event amplitude, duration and number of events over 40 trials (response probability) comparing values before and after tamoxifen (overexpression) for control (grey), gephyrin WT (dark blue), K148R (green), SSA (turquoise) and DN (pink) mutants. (**C**) Average percentage changes during spontaneous activity for calcium event amplitude, duration, and number of events over 40 trials (response probability) after gephyrin-mutant expression for individual neurons. The relative changes in Ca^2+^ transient amplitude, duration, and number of events of individual neurons by normalizing these parameters after gephyrin mutant expression to the activity before tamoxifen (TAM) injection [(After-Before)/Before]. Control, n = 491 neurons; gephyrin WT, n = 643 neurons; gephyrin-K148R, n = 308 neurons; gephyrin-SSA, n = 249 neurons; gephyrin-DN, n = 204 neurons; 3–4 mice per group. Statistics: linear mixed-effects models and Tukey post hoc tests. All bar graphs are represented as mean ± SEM. *p ≤ 0.05, **p ≤ 0.01, ***p ≤ 0.001.

**Figure 4:**
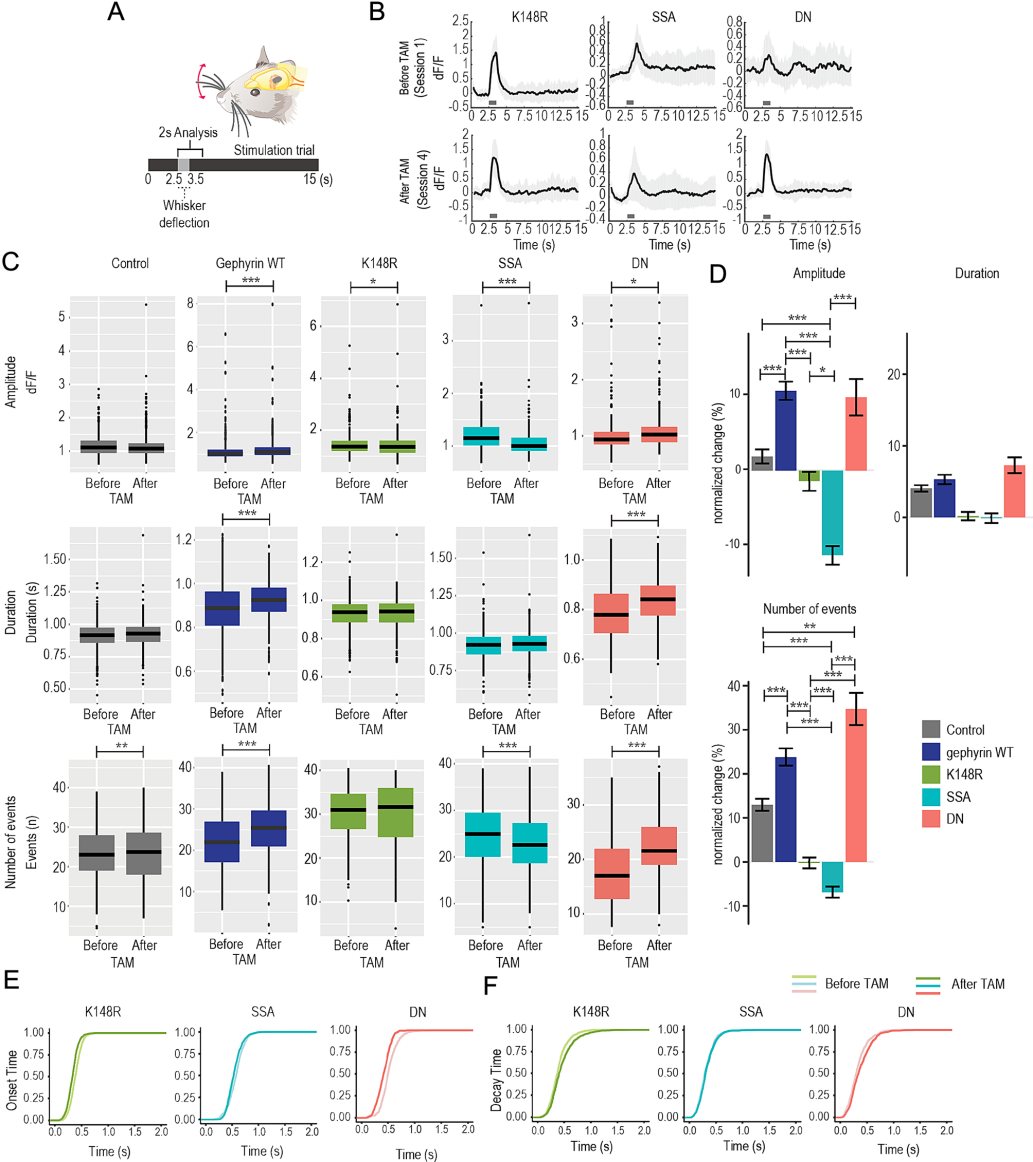
The expression of gephyrin mutants differentially modulates L2/3 pyramidal neuron excitability evoked by whisker stimulation. (**A**) Illustration of a trial with single whisker stimulation. (**B**) Average trace examples from imaging sessions for whisker stimulation trials (40 trials in one session) of both before and after gephyrin mutant expression. Grey bar: whisker stimulation. Lighter grey: standard deviation. (**C**) Calcium event amplitude, duration, and number of events (response probability) over 40 trials with whisker stimulation comparing values before and after tamoxifen for control (grey), gephyrin WT (dark blue), K148R (green), SSA (turquoise) and DN (pink) mutants. (**D**) Average percentage changes amplitude, duration and number of events after gephyrin-mutant expression for individual neurons during whisker stimulation. The relative changes in Ca^2+^ transient amplitude, duration, and number of events of individual neurons by normalizing these parameters after gephyrin mutant expression to the activity before tamoxifen (TAM) injection [(After-Before)/Before]. (**E**) Onset and (**F**) decay time for before and after expression of the gephyrin mutants. Control, n = 491 neurons; gephyrin WT, n = 643 neurons; gephyrin-K148R, n = 308 neurons; gephyrin-SSA, n = 249 neurons; gephyrin-DN, n = 204 neurons; 3–4 mice per group. Statistics: linear mixed-effects models and Tukey post hoc tests. All bar graphs are represented as mean ± SEM. *p ≤ 0.05, **p ≤ 0.01, ***p ≤ 0.001.

The functional impacts of gephyrin PTMs on cortical L2/3 pyramidal neurons were examined by using electrophysiology in acute slices (Fig. 2) and in vivo two-photon calcium imaging (Figs. 3, 4). For these functional assays, we injected AAV6-hSyn1-flex-eGFP-gephyrin variants, AAV6-CaMKIIα-ERT2-Cre and AAV6-CaMKIIα-RCaMP1.07 to co-express inducible eGFP-gephyrin variants and RCaMP1.07 in L2/3 principal cells in the mouse barrel cortex. After four weeks of virus co-expression, we injected tamoxifen (TAM) intraperitoneally (i.p) for four consecutive days to activate Cre recombinase and allow the expression of gephyrin mutant variants (Fig. 1B). We waited for a minimum of seven days, and we estimated of 30–40% gephyrin variant-positive L2/3 pyramidal neurons in a field of view after receiving TAM (Fig. 1C).

To study the effect of overexpression of gephyrin mutant variants, miniature inhibitory postsynaptic currents (mIPSC) from acute slice L2/3 pyramidal neurons were recorded from the eGFP-positive neurons, in the presence of tetrodotoxin (TTX), CNQX and AP-5. First, the expression of the gephyrin transgene with the K148R mutation had no effect on mIPSC amplitude compared to controls (28.8 ± 2.0 pA vs. 25.6 ± 1.8 pA; *P* = 0.641, Fig. 2A, B), nor was the inter-event interval (IEI) different, indicating the mIPSC frequency was unchanged (eGFP: 659.9 ± 78.3 ms vs. gephyrin-K148R: 408.0 ± 56.3 ms, Fig. 2C). This suggests that increased gephyrin clusters in the K148R group do not change spontaneous neurotransmitter release or the number of GABAergic inhibitory synapses. In contrast, the neurons expressing the gephyrin-SSA mutation had mIPSCs of increased amplitude (37.8 ± 2.7 pA; *P* = 0.026; Fig. 2A, B), likely as a result of increased GABA_A_R retention at synaptic sites^9^. The IEI was unchanged (eGFP: 659.9 ± 78.3 ms vs. gephyrin-SSA: 401 ± 73.7 ms, Fig. 2C), suggesting that there were no alterations in spontaneous neurotransmitter release. Neurons expressing the transgene gephyrin-DN which disrupts the gephyrin scaffold demonstrated reduced mIPSC amplitude (14.6 ± 1.6 pA) compared to GFP controls (28.8 ± 2.0 pA; *P* < 0.0001, Fig. 2B). The mIPSC inter-event interval (IEI) increased in gephyrin-DN neurons, indicating the frequency was reduced (inter-event intervals: 1451 ± 463.2 ms; GFP = 659.9 ± 78.3 ms; *P* = 0.038; Fig. 2C). Visually, the effect of gephyrin-DN on IEI had a bimodal distribution (Fig. 2C), but further analysis by D-Agostino-Pearsons test and Q-Q plot (Supplementary Fig. 1) determined the data was normally distributed. Notably, gephyrin-DN destabilizes synaptic GABA_A_Rs in vitro. It is plausible that a similar mechanism limits the availability of synaptic GABA_A_R in gephyrin-DN-expressing neurons.

Our electrophysiology results demonstrate that gephyrin mutants can influence GABAergic neurotransmission. Specifically, we found that gephyrin-SSA enhanced spontaneous inhibitory currents, while gephyrin-DN reduced spontaneous currents. Their effects on inhibitory transmission align with their known in vitro modifications of GABAergic synapses. Although it has been reported that K148R increases gephyrin clusters, its functional role is less clear in vivo. This emphasizes the significance of gephyrin and its PTMs in regulating pyramidal neuron GABAergic postsynaptic sites and their potential influence on overall neuronal activity. Importantly, it should be noted that the effects of PTMs are contingent upon specific contextual conditions.

### Changes in whisker stimulation-induced Ca^2+^ transients after gephyrin mutant expression

To understand how the gephyrin scaffold and PTMs differentially impinge on GABAergic neurotransmission in vivo, we measured changes in Ca^2+^ transients in the same L2/3 pyramidal neurons before and after gephyrin-mutant expression. We co-expressed the three viral vectors: AAV6-hSyn1-flex-eGFP-gephyrin variants, AAV6-CaMKIIα-ERT2-Cre and AAV6-CaMKIIα-RCaMP1.07 (Fig. 3A). Gephyrin-variant expression was controlled by tamoxifen-inducible Cre expression. As a control, we expressed RCaMP1.07 in a neighboring barrel area (Fig. 3A), but also compared the results from the gephyrin mutants to overexpression of gephyrin wildtype (WT; expressed in a neighboring barrel area). We examined all RCaMP-expressing neurons and compared changes in Ca^2+^ transients before and after eGFP-gephyrin expression. We analyzed two seconds from the onset of whisker stimulation (one-second stimulation period and one second afterward) to capture the stimulation-induced Ca^2+^ transient and the same period was analyzed in the spontaneous trials (Fig. 3A). The same neuronal populations within each field of view were imaged for multiple trials (40 spontaneous and 40 stimulation trials) for three repeated sessions in each condition (spontaneous vs. whisker stimulation-induced activities, before vs. after gephyrin-mutant expression). We considered the average activity of each neuron, across all trials and sessions for each condition.

First, we measured the spontaneous neuronal activity that occurred in trials without whisker deflection in the same period as the stimulus (2.5–4.5 s, Fig. 3A). The overall changes in amplitude, duration, and number of events (response probability) of Ca^2+^ transients were compared between before and after TAM administration (Fig. 3B). After tamoxifen, we observed an increase in the duration and number of events in the RCaMP control group, and an increase in amplitude in gephyrin WT overexpression control (Fig. 3B). The gephyrin- K148R mutant decreased the amplitude and duration of spontaneous Ca^2+^ events and decreased the response probability (the number of events in 40 trials; Fig. 3B, before vs. after TAM). The gephyrin-SSA mutant decreased the spontaneous Ca^2+^ event amplitude, without changing duration or response probability (Fig. 3B). The gephyrin-DN mutant showed a higher number of events (Fig. 3B). To better understand how individual neurons were impacted by gephyrin mutant expression and to better compare between mutants, we determined the relative changes in Ca^2+^ transient amplitude, duration, and number of events of individual neurons by normalizing these parameters after gephyrin mutant expression to the activity before tamoxifen (TAM) injection [(After-Before)/Before]. After normalization, no significant difference was observed between the control and gephyrin WT groups. Gephyrin WT had an increase in amplitude that was significant compared to K148R and DN (Fig. 3C). In comparison with the control and gephyrin WT, K148R displayed a reduction in spontaneous Ca^2+^ transient duration and SSA had reduced the number of events (Fig. 3C).

Whisker deflection evoked a Ca^2+^ response in all gephyrin mutants (Fig. 4A,B; example Ca^2+^ transient traces from a single imaging session). In the control group, the overall amplitude and duration did not change, but the number of events increased after TAM. Gephyrin WT, however, showed increases in all parameters after TAM. The gephyrin-K148R mutant had minimal effects on stimulus-evoked activity other than a slight decrease in Ca^2+^ amplitude (Fig. 4C), though this was not significant when comparing the normalized change after tamoxifen (Fig. 4D). The gephyrin-SSA mutant decreased the amplitude of evoked Ca^2+^ transients and the response probability (number of Ca^2+^ events across trials; Fig. 4C) to a greater degree than controls and the other mutants (Fig. 4D). The gephyrin-DN mutant slightly increased the Ca^2+^ amplitude (Fig. 4C), though this did not reach significance compared to the control group after tamoxifen normalization (Fig. 4D). The gephyrin-DN mutant also increased the Ca^2+^ event duration and the response probability (Fig. 4C,D). The effect of gephyrin variants on duration was not significant when compared to control and with other mutants (Fig. 4D). In addition, onset and decay times of the measured Ca^2+^ transients were similar before and after the expression of gephyrin variants (Fig. 4E,F). Taken together, the influence of gephyrin mutant variant expression on Ca^2+^ transient changes was consistent with their impact on inhibitory neurotransmission changes observed using the patch-clamp technique (Fig. 2): (i) gephyrin-K148R had minimal effects on mIPSCs and decreased spontaneous pyramidal neuron Ca^2+^, (ii) gephyrin-SSA increased mIPSC and decreased pyramidal neuron Ca^2+^ responses and (iii) gephyrin-DN decreased IPSCs and enhanced pyramidal neuron Ca^2+^. Notably, average neuronal responses were consistent between individual animals with the same mutant gephyrin expression (Supplementary Fig. 2).

### Gephyrin mutant expressions regulate the size of the responding neuronal population to whisker stimulation

**Figure 5:**
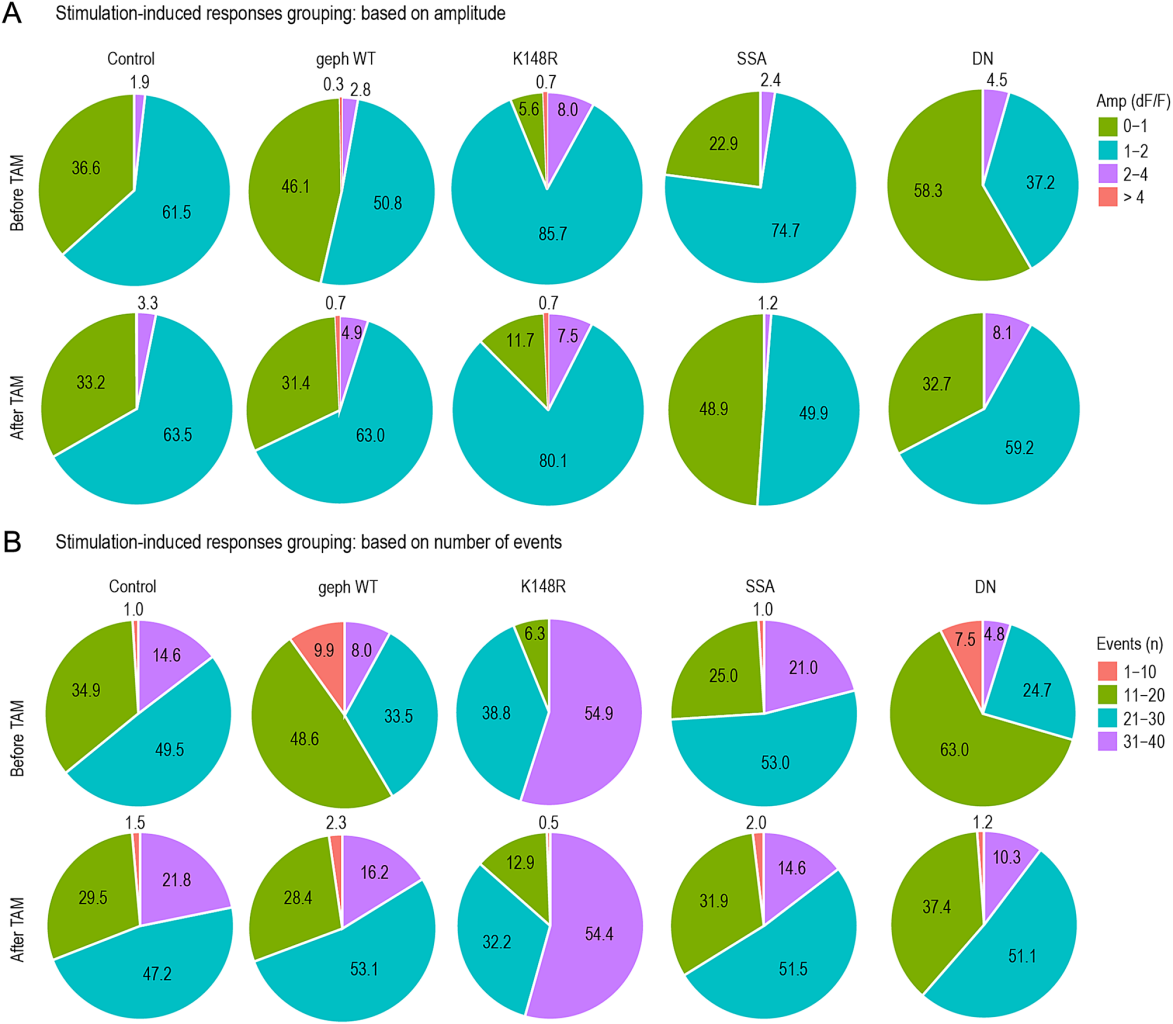
Spontaneous and whisker stimulation-induced activity changes in L2/3 pyramidal neurons expressing gephyrin variants. (**A**) Proportions of responding neurons before and after gephyrin-mutant expression in vivo are categorized based on Ca^2+^ transient amplitude. (**B**) Proportions of responding neurons before and after gephyrin-mutant expression are categorized based on the number of events (response probability). Control, n = 491 neurons; gephyrin WT, n = 643 neurons; gephyrin-K148R, n = 308 neurons; gephyrin-SSA, n = 249 neurons; gephyrin-DN, n = 204 neurons. TAM: tamoxifen. The numbers on the pie charts are rounded to one decimal place.

Sensory stimulation encoding in the L2/3 mouse barrel cortex is sparse due to strong GABAergic inhibition. Roughly 10% of the pyramidal neuron population reliably fires action potentials in response to whisker stimulation in every trial, which can be detected as robust Ca^2+^ events^18^. Therefore, we further examined the size of the responding neuronal population to our single-whisker stimulation paradigm before and after the expression of gephyrin PTM mutants. The neurons were grouped based on their Ca^2+^ transient amplitude (Fig. 5A) or their responding probability (number of events) (Fig. 5B). As expected, in control neurons, the amplitude and number of events within the populations remained comparable before and after tamoxifen injection (Fig. 5A,B). For gephyrin WT, cell population with higher amplitude and more number of events expanded. Similar to the control group, gephyrin-K148R mutant expression did not impact the population distribution based on amplitude or the number of events (Fig. 5A, B). Compared to before TAM, gephyrin-SSA mutant expression led to a smaller population of neurons responding with 1–2 dF/F and a larger population with 0–1 dF/F (Fig. 5A), but its effect on the number of Ca^2+^ events (i.e. the neuronal responsiveness) was minimal (Fig. 5B). In contrast, gephyrin-DN mutant expression reduced the low-amplitude population (0–1 dF/F) and increased the population of 1–2 dF/F amplitude neurons by more than 20% (Fig. 5A, green and turquoise). The expression of gephyrin-DN also shifted the population of neurons responding with 21–30 events/40 trials from 24.7 to 51.1% and 31–40 events/40 trials 4.8% to 10.3% (Fig. 5B). These shifts in population distributions for high responding neurons across different gephyrin mutants further support an enhancement in inhibitory transmission with gephyrin-SSA (fewer cells with large Ca^2+^ amplitudes) and a reduction in inhibitory transmission with gephyrin-DN (more cells with large Ca^2+^ amplitudes and more events in trials with stimulation). Overall, our data indicates that the gephyrin scaffold is an essential component for response strength and reliability to sensory inputs, and modulations of the gephyrin scaffold in pyramidal neurons were sufficient to fine-tune the population response to whisker stimulation.

### Gephyrin mutants show a preference for GABA_A_R subtypes

**Figure 6:**
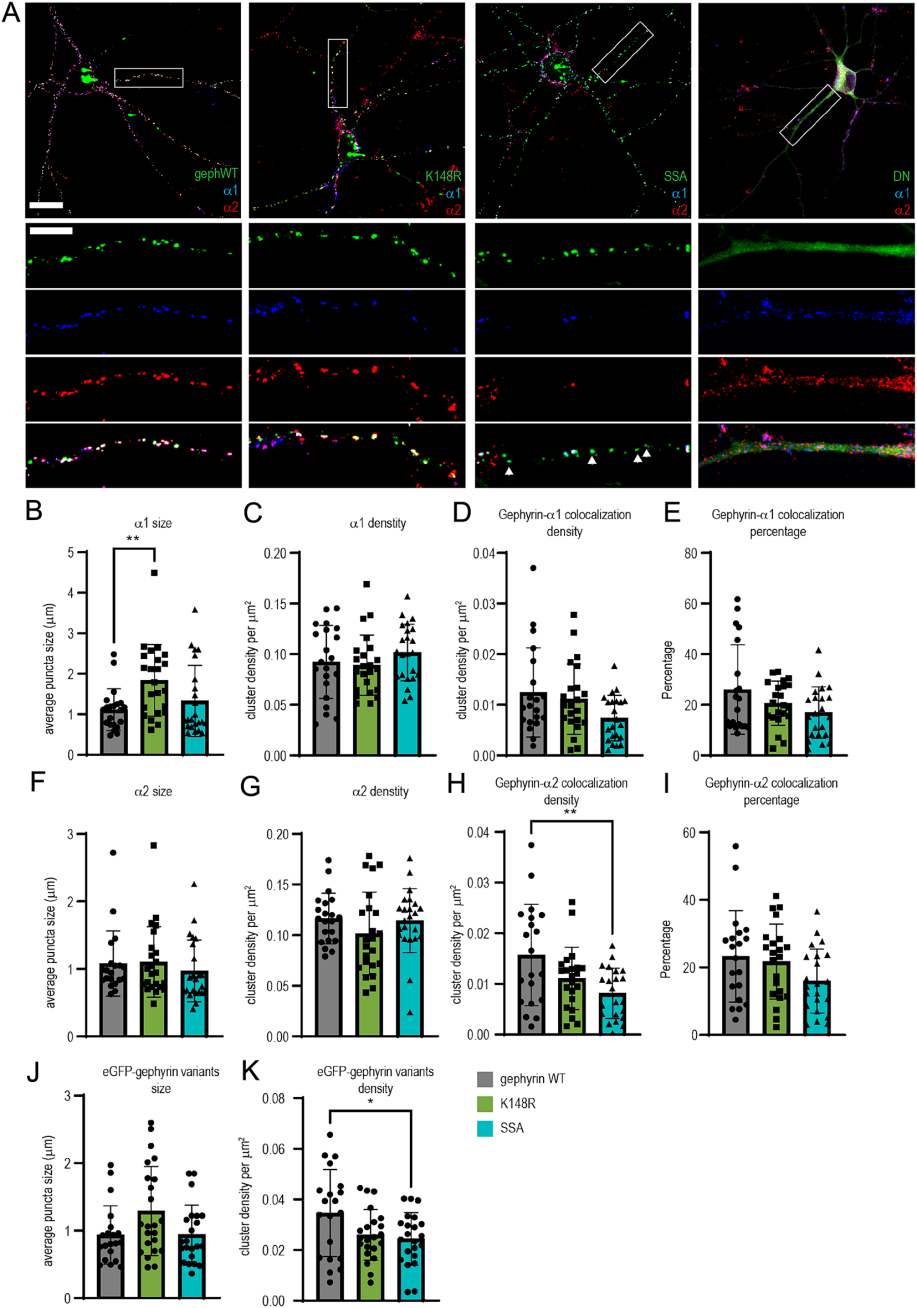
Interaction of gephyrin variants and synaptic α1- or α2-GABA_A_R subtypes. The primary cortical neurons were transfected with eGFP-gephyrin WT, eGFP-gephyrin DN, eGFP-Gephyrin K148R or eGFP-gephyrin SSA mutant at 13 days in vitro (13 DIV) and stained for α1 and α2 subunits of GABA_A_Rs at 20 DIV. (**A**) Example images of transfected neurons with gephyrin mutants. Lower panels: magnified images of selected dendrites (white box). Scale bar: 25 μm in full images, 10 μm in zoom-in images. White arrows: eGFP-gephyrin clusters without GABRA2 staining. (**B**–**E**) Cluster analysis was performed to show the size, density, and colocalization of eGFP-gephyrin variants with α1-GABA_A_Rs. (**F**–**I**) Cluster analysis was performed to show the size, density and colocalization of eGFP-gephyrin variants with α2-GABA_A_Rs. eGFP-gephyrin DN was excluded from cluster analysis as the GFP signal and the staining of α1-GABA_A_Rs and α2-GABA_A_Rs were diffused. (**J**–**K**) eGFP-gephyrin variant size and density. N = 20–22 neurons per group, 3 independent experiments. Statistics: One-way ANOVA, with Tukey post hoc test. *p ≤ 0.05, **p ≤ 0.01, ***p ≤ 0.001.

In vitro studies have identified the gephyrin scaffold as a signaling hub at GABAergic postsynaptic sites to facilitate activity-dependent synaptic GABA_A_R recruitment and removal. The involvement of synaptic GABA_A_R can be a potential mechanism that leads to the functional changes we have observed when gephyrin mutants are expressed in pyramidal neurons. However, it remains unclear how specific PTMs on gephyrin alter its ability to recruit either α1- or α2-containing GABA_A_Rs, which are important synaptic GABA_A_R subtypes in L2/3 cortical circuits. We used cortical primary neuron cultures and examined the co-localization of α1 or α2 subunits with eGFP-gephyrin mutants by using cluster analysis. We transfected primary cortical neurons with either eGFP-gephyrin (WT), eGFP-gephyrin K148R, eGFP-gephyrin SSA, or eGFP-gephyrin DN at 13 days in vitro (13 DIV). At 20 DIV, the cultures were co-stained for α1 and α2 GABA_A_R subunits (Fig. 6A). The eGFP-gephyrin or its mutant variants were co-labelled with α1 and α2 GABA_A_Rs. Gephyrin mutant variants formed defined clusters in the primary neurons and exhibited phenotypes consistent with earlier reports^11,14^, except the DN mutant that showed diffuse signals across soma and dendrites. The DN-expressing neurons also showed diffusely labeled α1-and α2-GABA_A_Rs, which led to difficulty in cluster analysis. Therefore, it was excluded from the quantitative comparisons.

We examined the size and density of α1 and α2 subunits of GABA_A_R and their colocalization density and percentage with the gephyrin variants (Fig. 6B–I). The neurons expressing K148R mutant gephyrin had larger α1-GABA_A_R clusters (Fig. 6B), but the overall density of α1-GABA_A_R clusters was not altered in neurons expressing either WT or mutant eGFP-gephyrin (Fig. 6C). Colocalization of WT, K148R, and SSA mutant gephyrin with α1- GABA_A_Rs was not significantly different (Fig. 6D, E). The cluster size and density of α2-GABA_A_R clusters were similar in neurons expressing either eGFP-gephyrin WT, SSA, or K148R (Fig. 6F, G). However, the SSA mutant exhibited significantly reduced colocalization density with α2-GABA_A_Rs (Fig. 6H) and a tendency for lower colocalization percentage (Fig. 6I). Analysis of eGFP-gephyrin clusters showed that no change in cluster size (Fig. 6J) and SSA had lower cluster density (Fig. 6K). This reduction in density seems to affect the colocalization of α1-GABAARs (trend, Fig. 6D) and α2-GABA_A_Rs (significant, Fig. 6H). Overall, our results highlight the significance of different post-translational modifications on gephyrin for the recruitment of specific GABA_A_R subtypes to facilitate cortical circuit function. These changes in the synaptic localization of α1- or α2-GABA_A_R subtypes may explain the differences in electrophysiology and population Ca^2+^ dynamics that we observed above.

## Discussion

The neuronal response to different interneuron inputs relies heavily on the downstream receptor complex and postsynaptic density proteins, suggesting neurotransmission can be regulated at the GABAergic postsynaptic site. While it is well established that GABAergic inhibition plays a central role in sparse sensory processing, the contribution of GABAergic postsynaptic components to the functional specificity of the somatosensory cortex microcircuit is unknown. To fill this knowledge gap, we present evidence showing the central role for gephyrin scaffolding during sensory stimulation and fine-tuning L2/3 pyramidal neuron activity, potentially via selective recruitment of GABA_A_R subtypes to inhibitory postsynaptic sites. This is the first in vivo study of the impact of gephyrin on intact circuits.

As summarized in Table 2, mutants that target specific gephyrin PTMs have differential effects on mIPSCs and pyramidal neuron Ca^2+^ responses in vivo. The gephyrin-K148R SUMO1 conjugation mutant did not have a significant impact on pyramidal neuron inhibition overall but decreased the duration of spontaneous Ca^2+^ activity (Fig. 3B, C) and enlarged α1 subunit clusters (Fig. 6B). Increased GABA_A_Rs clustering in vitro fits with previous studies of this mutant^14^, but we expected this would enhance neuronal inhibition in vivo*.* It is possible that we did not observe strong effects on pyramidal activity because gephyrin-K148R expression was induced in adult mice compared to earlier developmental stages in previous work^14^. The gephyrin-SSA (S303/S305) phosphorylation mutant increased mIPSC amplitude (Fig. 2) and in vivo imaging data showed a decrease in Ca^2+^ amplitude, number of events (Figs. 3, 4), and a less excitable neuronal population to whisker stimulation (Fig. 5). Quantum dot–based single-GABA_A_R tracking studies have shown that the gephyrin scaffold influences GABA_A_R surface mobility and synapse retention^31^. The gephyrin-SSA mutant increases the synaptic confinement of α2-GABA_A_Rs in response to 4-Aminopyridine-induced circuit activation^9^ which is in line with our results. Increased synaptic confinement of GABA_A_Rs in gephyrin-SSA-expressing neurons could disrupt dynamic shifts in reducing inhibitory synapses according to excitatory inputs to the neuron. Lastly, we observed an increase in mIPSC IEI and reduced amplitude upon gephyrin-DN mutant expression in our electrophysiology studies (Fig. 2). In vivo imaging data demonstrated that gephyrin-DN mutant expression increases the Ca^2+^ events (Figs. 3,4) and expands the neuronal population of high responders after whisker stimulation by more than 20% (Fig. 5). Likely, the gephyrin-DN mutant disrupted the gephyrin scaffold and this increased diffusion and exploration of GABA_A_Rs at both synaptic and extrasynaptic sites^9^. Overall, our data implicate a dynamic recruitment model for specific GABA_A_R subtypes based on gephyrin PTMs. It is conceivable that signaling cascades, possibly induced by specific demand, cause gephyrin PTMs that switch affinity towards specific GABA_A_R subtypes for synapse recruitment. Further experiments are needed to address the involvement of other GABA_A_ receptor subtypes and binding proteins downstream of gephyrin clusters.

**Table 2:** Summary of the effects of different mutants used in this study.

Mutation	IPSCs	Ca^2+^ events	GABA_A_R subunits	Conclusion
Gephyrin K148R	No change in mIPSCs	↓ Spontaneous duration	↑ α1 clusters	Minor effect on inhibition
Gephryin SSA	↑ mIPSC	↓ Amplitude	↓ α2 colocalization at baseline	↑ Inhibition
		↓ # Events		
Gephryin DN	↓ mIPSCs	↑ # of events, ↑ amplitude	↓ α1 & α2 clusters	↓ Inhibition
	↓ Evoked IPSCs			

It should be noted that we observed some changes in RCaMP controls and gephyrin WT overexpression groups (Figs. 3B, 4B) after tamoxifen that suggested an increase in pyramidal neuron activity. This potentially occurred because these groups were located in a neighboring whisker barrel column close to the mutant gephyrin-expressing cells. As adjacent barrels have some reciprocal functional connectivity^32,33^, and it is likely that gephyrin mutant expression also affect neighboring barrel circuits due to changes in inhibition. Therefore, these changes in our controls could be caused by mutant gephyrin effects in the neighboring gephyrin mutant barrel. In the case of gephyrin WT, the enhanced neuronal Ca^2+^ amplitude and response probability during whisker stimulation (Fig. 4) was similar to the gephyrin- DN mutant. This suggests that overexpression of wildtype gephyrin in vivo likely reaches high levels that interfere with normal GABAergic signaling. Unfortunately, it was not possible to measure the expression level of gephyrin WT, nor the post-translational modifications or processing of the overexpressed gephyrin WT proteins within the cells. Therefore, the variability in cell responses may stem from differing levels of overexpression within the cells. Nevertheless, our results with specific gephyrin mutants K148R and SSA are different and unique to each of the mutants (Figs. 2, 3, 4), suggesting that the observed effects on pyramidal neuron activity are due to the mutant gephyrin interactions and not strictly due to protein overexpression.

### Gephyrin PTM and their preference of GABA_A_R subtypes

In the cortex, the synaptic GABA_A_ receptor (GABA_A_Rs) subtypes express preferentially within specific cortical layers^34^. The α1 subunit-containing GABA_A_Rs (α1-GABA_A_Rs) are uniformly distributed across all six cortical layers, whereas the α2 subunit-containing GABA_A_Rs (α2-GABA_A_Rs) are abundantly expressed in the supragranular layers (layer 1, 2 and 3), and the α3 subunit containing-GABA_A_Rs are more abundant in layers five and six^26^. This spatial segregation of GABA_A_R subtypes implies that distinct functional roles exist for GABA_A_R subtypes within the cortex. We tested gephyrin K148R, SSA, and DN mutants in primary cortical neuronal culture to compare their preference on common synaptic GABA_A_R subtypes, α1- and α2-containing GABA_A_Rs. The expression of the gephyrin K148R mutant decreased spontaneous Ca^2+^ event duration and increased α1- cluster size (Figs. 3, 6), which implies that α1-GABA_A_Rs may contribute to synaptic inhibitory tone within the microcircuit that influences the response in pyramidal neurons during spontaneous circuit activity. In the gephyrin-SSA mutant, we observed a decrease in the number of Ca^2+^ amplitude and events particularly during whisker stimulation (Figs. 3, 4), which may occur because of increased α2-GABA_A_R retention at synaptic sites^9^. The α2-GABA_A_Rs are distributed in upper cortical layers in rodents^26^ and therefore the functional effects of SSA observed in this study might be specific for L2/3. The expression of SSA in primary neuron culture, however, showed a reduction in α2-GABA_A_R colocalization. The discrepancy in our in vitro result likely arises from the fact that our primary culture was without external stimulation. Therefore, it is possible that gephyrin PTMs may behave differently depending on the context, and this further suggests the involvement of other proteins that may be associated with gephyrin or receptors.

Whether any specificity exists between a GABA_A_R subtype and a subgroup of interneurons is highly debatable. Depending on the study, α1-GABA_A_Rs have been localized at PV+ basket cell terminals, α2-GABA_A_Rs have been localized at CCK+ cell terminals^35,36^ and α1-and α2-GABA_A_R subtypes have been localized at both CCK+ and PV+ terminals^37^. The exact distribution of specific α-subunit containing GABA_A_Rs to distinct synaptic sites is further complicated by the homomeric and heteromeric nature of these receptor subtypes. Both α1- α1 homomers and α1- α2 heteromers have been identified^38–40^. Hence, our identification of gephyrin PTM as a determinant for GABA_A_R synapse recruitment adds an additional layer of complexity to the understanding of GABA_A_R subtypes and circuit activity.

### Gephyrin-mediated activity adaptations within L2/3 microcircuit

L2/3 pyramidal neurons have more hyperpolarized resting potentials than deeper cortical layers; therefore, they require more excitatory input and/or reduced GABAergic input to overcome the action potential threshold^20^. We obtained up to 40% co-expression of gephyrin mutants with RCaMP and Cre within the L2/3 pyramidal neurons. Despite this small-scale co-infectivity ratio, we successfully perturbed microcircuit function upon gephyrin-mutant expression in the barrel cortex. The effects of gephyrin mutant expression in a small number of pyramidal neurons are likely to be amplified by lateral excitation within the L2/3. Similarly, the local inhibitory neurons are likely to be affected indirectly by the activity change of the pyramidal neurons. Future experiments focusing on the activity of specific interneuron subtypes in gephyrin mutants are needed to gain a full picture of the microcircuit alterations upon changes in gephyrin clustering.

It is currently hypothesized that both Hebbian and non-Hebbian synaptic plasticity mechanisms might underlie the strengthening and weakening of cellular responses after whisker stimulation and/or trimming^18,41–43^ By altering gephyrin PTMs, we observed both the strengthening and weakening of individual pyramidal neuron activity, which modulated the size of the responding neuronal population to single-whisker stimulation. Disruption of the gephyrin scaffold through the expression of the gephyrin-DN-mutant led to an expansion in neuronal populations with higher amplitude in Ca^2+^ transients. On the other hand, expression of the gephyrin-SSA mutant led to a decrease in the number of high responder cells (Fig. 5). These results support Hebbian synaptic plasticity, i.e. that cells with increased/decreased activity are more likely to strengthen/weaken the activity of their synaptic partners. It is tempting to speculate that the gephyrin scaffold has the potential to regulate Hebbian plasticity via its PTMs, and future studies of gephyrin clustering in homeostatic plasticity between pyramidal-pyramidal neurons and interneuron-pyramidal neurons will help to clarify this role.

### Limitations

This study possesses several inherent limitations that merit acknowledgment. Firstly, the inability to distinguish between mutant gephyrin and endogenous gephyrin expression poses a challenge, leaving us uncertain about whether overexpression influenced endogenous gephyrin levels. While the dominant effects of gephyrin mutants have been evident in primary neuron culture, the complexity of the in vivo environment introduces additional nuances. Although our findings indicate distinct effects of each gephyrin mutant on pyramidal neuron activity, it remains plausible that endogenous gephyrin may compensate for certain alterations in GABAergic activity. Secondly, our decision to exclusively use female mice aimed at averting aggression and minimizing cranial window damage during group housing introduces a potential limitation concerning sex differences in our findings. Thirdly, variations in neuronal populations between mutants before tamoxifen administration were observed (Fig. 5), potentially attributable to leaky CreERT2 activity. This phenomenon might have obscured differences between cells before and after tamoxifen administration. Lastly, the experiments were conducted in anesthetized mice, and it is well-known that anesthesia dampens neuronal activity. We anticipate that neuronal responses to whisker stimulation would be more robust in awake mice, offering a clearer perspective on the impact of gephyrin PTM mutants on circuit activity. Despite controlling for the level of isoflurane, variations in the depth of anesthesia experienced by mice across imaging sessions could contribute to the observed data variability. These limitations underscore the need for cautious interpretation and highlight avenues for refining experimental design in future investigations.

## Conclusion

The regulatory role of GABAergic interneurons in modulating the excitability of layer 2/3 (L2/3) pyramidal neurons in the barrel cortex during sensory encoding is well established. In our study, we introduce new evidence that this regulation is intricately influenced by the GABA_A_R scaffolding protein gephyrin. Through specific post-translational modifications, gephyrin demonstrates the capacity to both enhance and diminish pyramidal neuron activity, consequently impacting sensory encoding. This discovery unveils new avenues for understanding the molecular components operating at GABAergic postsynaptic terminals, offering insights into their potential to guide and govern microcircuit activity.

### Supplementary Information

Supplementary Figures.

## Data Availability

Data tables analysed during the current study are available from the corresponding author upon reasonable request.
