# Supplementary material for: The gephyrin scaffold modulates cortical layer 2/3 pyramidal neuron responsiveness to single whisker stimulation
Source: Sci Rep. 2024 Feb 20;14:4169. doi: 10.1038/s41598-024-54720-7 (PMC10879104; doi:10.1038/s41598-024-54720-7)
Supplement: Supplementary file 1 — Supplementary Figures. [file 41598_2024_54720_MOESM1_ESM.pdf]

Supplementary Material

The gephyrin scaffold modulates cortical layer 2/3 pyramidal neuron responsiveness to single whisker stimulation

Yuan-Chen Tsai[1,2], Mohammad Hleihil[1,2], Kanako Otomo[1], Andrin Abegg[1], Anna Cavaccini[3], Patrizia Panzanelli[4], Teresa Cramer[1,2], Kim David Ferrari[1,2], Matthew J.P. Barrett[1,2], Giovanna Bosshard[1], Theofanis Karayannis[3], Bruno Weber[1,2], Shiva K. Tyagarajan[1,2,†], Jillian L. Stobart[1,5,†,*]

† These authors contributed equally to this work and share senior authorship.

***Correspondence:**

Corresponding Author: Jillian.Stobart@umanitoba.ca

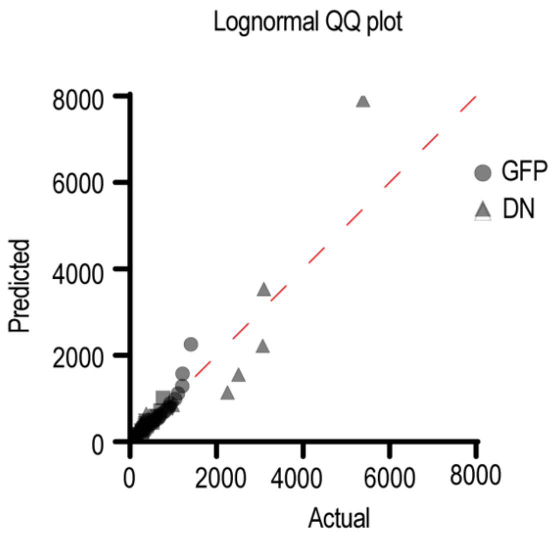

Supplementary Figure 1. Log normal Q-Q plot for IEI data from controls (GFP) and gephyrin-DN mutant. Data was found to be normally distributed.

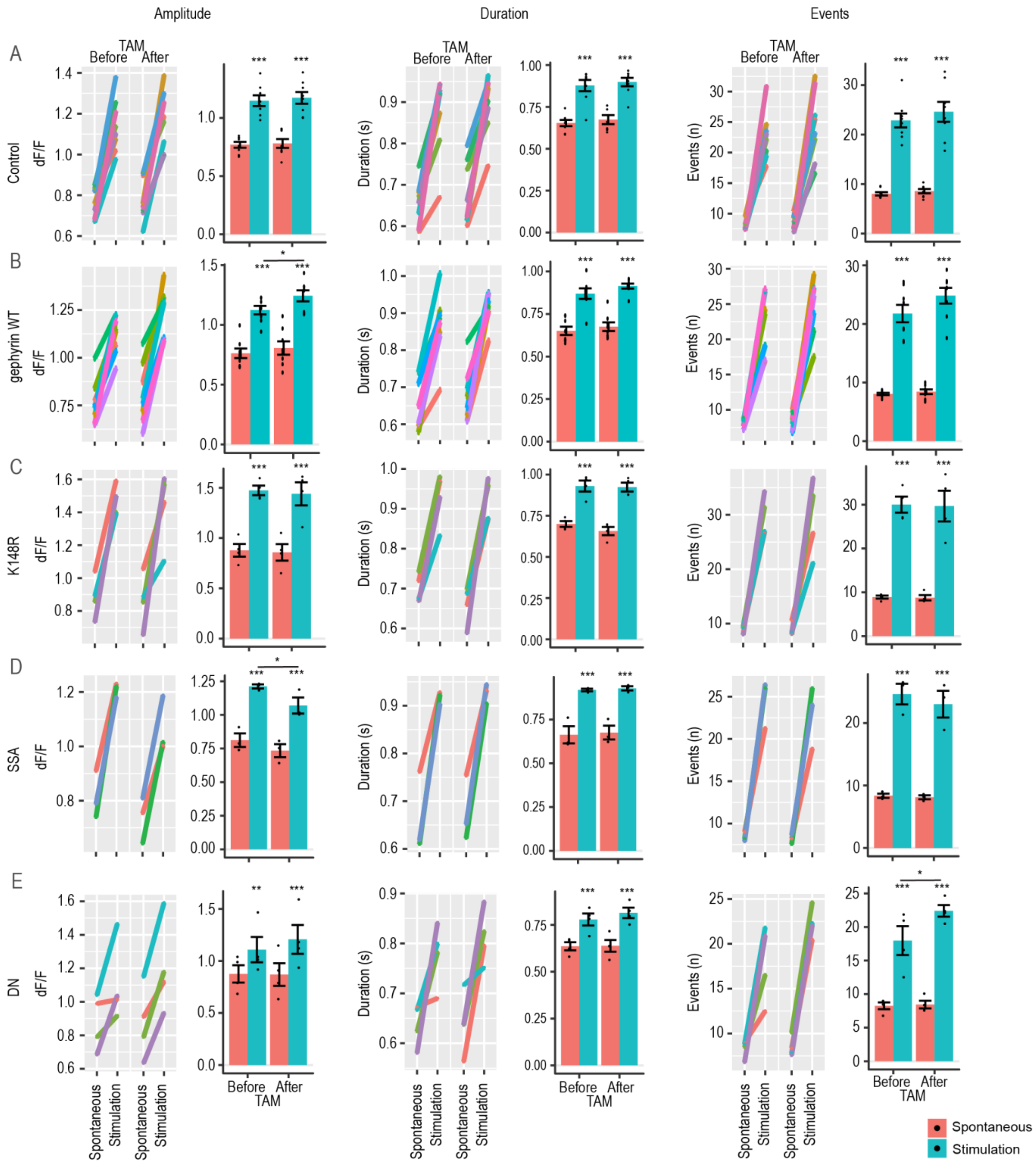

Supplementary Figure 2. Averaged spontaneous or whisker stimulation-induced Ca^{2+} transient amplitude, duration, and number of events before and after TAM injection in control **(A),** gephyrin

WT **(B)**, K148R **(C),** SSA **(D)** and DN **(E)** groups. Each line/dot: one animal. Number of neurons measured: control, n=491 neurons, 8 animals; gephyrin WT, n=643, 8 animals, gephyrin-K148R, n=308 neurons, 4 animals; gephyrin-SSA, n=249 neurons, 3 animals; gephyrin-DN, n=204 neurons, 4 animals. Statistics: linear mixed-effects models and Tukey post hoc tests. All bar graphs are represented as mean ± SEM. *$p \leq 0.05$, **$p \leq 0.01$, ***$p \leq 0.001$.
